# Anesthesia Consideration for a Patient With Incidentally Diagnosed Anomalous Origin of Right Coronary Artery Originating From Pulmonary Trunk (ARCAPA): A Case Study

**DOI:** 10.7759/cureus.28955

**Published:** 2022-09-08

**Authors:** Dylan S Irvine, Kristina Rozava, Alexander Theodotou, Raymond Evans, Jeffrey Huang

**Affiliations:** 1 Medical School, Nova Southeastern University Dr. Kiran C. Patel College of Osteopathic Medicine, Davie, USA; 2 Medical School, University of South Florida Health Morsani College of Medicine, Tampa, USA; 3 Medical School, University of South Florida Heath Morsani College of Medicine, Tampa, USA; 4 Anesthesiology, Moffitt Cancer Center, Tampa, USA; 5 Anesthesiology, Health Outcomes and Behavior, Moffitt Cancer Center, Tampa, USA

**Keywords:** surgical management, surgery, anesthesiology, anomalous origin of coronary artery, arcapa

## Abstract

Anomalous origin of the right coronary artery originating from the pulmonary trunk (ARCAPA) is a rare congenital coronary anomaly that is usually diagnosed incidentally. Although usually asymptomatic, ARCAPA can lead to myocardial ischemia of the left ventricular wall and/or sudden cardiac arrest. Here, we report the case of a 48-year-old female who presented for recurrent malignant pleural effusion, who was scheduled for a bronchoscopy, thoracoscopic evaluation of left pleural effusion, multiple excisional biopsies of the left chest wall and costophrenic parietal pleural nodules, and insertion of tunneled PleurX™ catheter (Becton, Dickinson and Company, Franklin Lakes, New Jersey, United States). ARCAPA was discovered incidentally in this patient during the preoperative evaluation. The patient was asymptomatic and echocardiogram findings were within normal limits. No additional intervention was required, and the patient was managed satisfactorily with general anesthesia.

## Introduction

Anomalous right coronary artery originating from the pulmonary artery (ARCAPA) is a rare congenital coronary artery that is typically asymptomatic and has a prevalence of about 0.002% within the general population [1-2]. In contrast to patients who present with anomalous left coronary artery from the pulmonary artery (ALCAPA), who usually present in infancy with signs of congestive heart failure and left ventricular ischemia, patients with ARCAPA are commonly asymptomatic and diagnosed incidentally during childhood [1,3]. The incidence of ALCAPA is higher than that of ARCAPA, likely due to the closer proximity of the left coronary bud to the pulmonary artery sinus [3]. While usually isolated, other anomalies such as tetralogy of Fallot, septal defects, and aortopulmonary window have been reported in up to a third of ARCAPA patients [1].

## Case presentation

We report the case of a 48-year-old female patient who was incidentally diagnosed with ARCAPA during routine preoperative investigation. She presented for evaluation of malignant recurrent left pleural effusion and was subsequently scheduled for a bronchoscopy, thoracoscopic evaluation of left pleural effusion, multiple excisional biopsies of left chest wall and costophrenic parietal pleural nodules, and insertion of tunneled PleurX™ catheter (Becton, Dickinson and Company, Franklin Lakes, New Jersey, United States).

Past medical history was significant for left-sided breast carcinoma. Airway and cardiopulmonary examinations were normal. EKG showed normal sinus rhythm. The patient had Holter monitoring three months prior due to palpitations. It showed a predominant sinus rhythm with a heart rate of 85. During routine preoperative investigation, echocardiography showed an incidental diagnosis of ARCAPA. Echocardiogram results are summarized in Table 1. 

**Table 1:** Summary of routine preoperative echocardiogram results.

Echocardiogram Findings	
Left Ventricle Size	Normal
Left Ventricle Wall Dimensions	Normal
Left Ventricle Systolic Function	Normal
Left Ventricle Diastolic Function	Normal
Estimated Left Ventricle Ejection Fraction (EF)	55-60% (Normal)
Right Ventricle Size	Normal

The patient's medications included midodrine and benzonate. Allergies for codeine, penicillin, and midazolam were noted. She also had multiple laparoscopic surgeries without anesthetic complications. A cardiologist was consulted and concluded that since the patient's ARCAPA was asymptomatic, no further intervention was required at this time.

General anesthesia was induced with fentanyl, propofol (2 mg/kg), and rocuronium. A size 35F left-sided double-lumen tube (DLT) was inserted without difficulty. The placement of the DLT was confirmed using bronchoscopy, which demonstrated no abnormal findings. A radial artery line was inserted and the FloTrac™ sensor (ver.4.00, Edwards Lifesciences, Irvine, California, United States) was connected. The cardiac output (CO), cardiac index (CI), stroke volume (SV), and stroke volume variation (SVV) were evaluated using the EV 1000 monitor, and there were no abnormalities.

Anesthesia was maintained with inhaled sevoflurane and intravenous rocuronium. During the surgery, the hemodynamic and respiratory statuses were stable. Her mean arterial pressure (MAP) was maintained at 100 mmHg, heart rate 80 beats per minute, peripheral capillary oxygen saturation (SaO2) 100%, and temperature 36.5^o^C. Arterial blood gas findings were normal. Her EKG showed normal sinus rhythm throughout the case. She was extubated at the end of the surgery and transferred to the post-anesthetic care unit (PACU). The rest of the hospital course was uneventful. She was discharged home on postoperative day one.

## Discussion

The incidence of congenital coronary artery anomalies is very rare, with an estimated incidence of 0.3-0.9% [4]. Among anomalous origin from the pulmonary artery, four variations, including ALCAPA, ARCAPA, origin of an accessory coronary artery from the pulmonary artery, and origin of the entire coronary circulation from the pulmonary artery, have been described [5].

ARCAPA is a congenital coronary anomaly that is most commonly asymptomatic and diagnosed incidentally in most patients during other evaluations, such as conventional angiography, which is used to work up other cardiac conditions [1,6]. Although conventional angiography is commonly utilized to diagnose coronary anomalies, noninvasive modalities such as cardiac computed tomography or cardiac magnetic resonance imaging can also be utilized to provide anatomic information on aberrant coronary vessels [7]. ARCAPA has been identified in patients ranging from birth to over 90 years of age [1-2]. The estimated incidence of ARCAPA is 0.002% within the general population, however, because patients are most commonly asymptomatic, the true incidence could be higher [1,8]. ARCAPA is less likely to be fatal, relative to ALCAPA [9].

Although ARCAPA is most commonly asymptomatic, clinical presentation of symptomatic patients is highly variable and is largely associated with the underlying pathophysiology [2]. When symptomatic, presentation varies and may include dyspnea (17%), fatigue (13%), congestive heart failure (30%), myocardial infarction (9%), and sudden cardiac arrest (17%) [8]. It has been suggested that the age of onset and the nature of the symptomatology present can be predicted by the direction of filling in the anomalous vessel, the formation of collateral vessels with the left coronary system, as well as the significance of the resulting left to right shunt [6]. Once ARCAPA is identified, surgical correction is recommended to reduce the risk of sudden cardiac death as well as other potentially fatal outcomes [6]. Correction can be made through re-implanting the anomalous right coronary artery to the aorta, or through ligation [10]. Conservative measures or withholding correction altogether for this condition have been described when the risk of surgery is deemed to outweigh the benefits [11]. This was a decision made in the patient we described in this case, as she was currently asymptomatic and had other comorbidities that increased operative risk. Her preoperative evaluation included a repeat echocardiogram to assess cardiac function. An arterial line was inserted to monitor her blood pressure and opioids were used to block sympathetic stimulation causing reduced coronary blood perfusion.

Anesthetic and surgical considerations must be considered in cases with anomalous coronary arteries. Anesthetic considerations in the management of patients with anomalous aortic origin of coronary artery (AAOCA) are outlined in a review by Kloesel et al. (2018) [12]. These include preoperative considerations such as patient history and review of cardiac testing and monitoring with standard ASA monitors and five-lead ECG, avoiding hypertension, hypotension and tachycardia during induction, and reducing the potential for stress and tachycardia in both maintenance and through emergence [12]. Anesthetic management during the repair of ALCAPA must be taken with great care due to the infant’s significantly increased risk of myocardial ischemia, cardiac arrest, and sudden cardiac death [13]. It has been recommended that intravenous induction and maintenance anesthetic should be attempted if possible [13]. In both ALCAPA and ARCAPA surgical correction, safe perioperative anesthetic management requires an understanding of myocardial blood flow and the coronary steal phenomenon [13-14]. The coronary steal phenomenon states that vasodilation of a segment of myocardial vasculature can take blood from another segment [13-14]. In the repair of ARCAPA, ventricular ischemia due to coronary steal may be worsened by the aortic runoff due to the aortopulmonary window [15]. Thus, careful anesthetic consideration of myocardial blood flow must be considered [15].

## Conclusions

This report describes the incidental diagnosis of ARCAPA, a rare coronary anomaly in a patient who presented with recurrent malignant pleural effusion. She was scheduled for a bronchoscopy, thoracoscopic evaluation of left pleural effusion, multiple excisional biopsies of left chest wall and costophrenic parietal pleural nodules, and insertion of tunneled PleurX catheter. Although the management strategy of surgical correction versus conservative measures and observations varies depending on the clinical scenario on a case-by-case basis, knowledge of the presence of this condition is important so perioperative anesthetic considerations can be made and the risk of sudden cardiac death and other comorbidities can be avoided due to the potential of coronary steal phenomenon from the anomalous myocardial blood flow.
